# Impact of Chronic Tetracycline Exposure on Human Intestinal Microbiota in a Continuous Flow Bioreactor Model

**DOI:** 10.3390/antibiotics10080886

**Published:** 2021-07-21

**Authors:** Youngbeom Ahn, Ji Young Jung, Ohgew Kweon, Brian T. Veach, Sangeeta Khare, Kuppan Gokulan, Silvia A. Piñeiro, Carl E. Cerniglia

**Affiliations:** 1 Division of Microbiology, National Center for Toxicological Research, U.S. Food and Drug Administration, Jefferson, AR 72079, USA; jyjung@nnibr.re.kr (J.Y.J.); oh-gew.kweon@fda.hhs.gov (O.K.); sangeeta.khare@fda.hhs.gov (S.K.); kuppan.gokulan@fda.hhs.gov (K.G.); carl.cerniglia@fda.hhs.gov (C.E.C.); 2 Office of Regulatory Affairs, Arkansas Laboratory, U.S. Food and Drug Administration, Jefferson, AR 72079, USA; brian.veach@fda.hhs.gov; 3 Division of Human Food Safety, Center for Veterinary Medicine, U.S. Food and Drug Administration, Rockville, MD 72079, USA; Silvia.Pineiro@fda.hhs.gov

**Keywords:** tetracycline, chronic exposure, human intestinal microbiota, continuous flow bioreactor model

## Abstract

Studying potential dietary exposure to antimicrobial drug residues via meat and dairy products is essential to ensure human health and consumer safety. When studying how antimicrobial residues in food impact the development of antimicrobial drug resistance and disrupt normal bacteria community structure in the intestine, there are diverse methodological challenges to overcome. In this study, traditional cultures and molecular analysis techniques were used to determine the effects of tetracycline at chronic subinhibitory exposure levels on human intestinal microbiota using an in vitro continuous flow bioreactor. Six bioreactor culture vessels containing human fecal suspensions were maintained at 37 °C for 7 days. After a steady state was achieved, the suspensions were dosed with 0, 0.015, 0.15, 1.5, 15, or 150 µg/mL tetracycline, respectively. Exposure to 150 µg/mL tetracycline resulted in a decrease of total anaerobic bacteria from 1.9 × 10^7^ ± 0.3 × 10^7^ down to 2 × 10^6^ ± 0.8 × 10^6^ CFU/mL. Dose-dependent effects of tetracycline were noted for perturbations of *tetB* and *tetD* gene expression and changes in acetate and propionate concentrations. Although no-observed-adverse-effect concentrations differed, depending on the traditional cultures and the molecular analysis techniques used, this in vitro continuous flow bioreactor study contributes to the knowledge base regarding the impact of chronic exposure of tetracycline on human intestinal microbiota.

## 1. Introduction

The gut microbial community plays an important role in protecting a host against invading pathogens by regulating host immunity as well as metabolic processes while also carrying out physiological functions such as strengthening gut integrity or shaping the intestinal epithelium [1,2,3]. Research studies with drugs used in human medicine indicate that therapeutic levels of antibiotics can disrupt intestinal microbiota composition and induce the selection of antimicrobial-resistant strains. Antimicrobial drugs are also known to alter metabolic activity of the microbiota as well as change colonization resistance properties of the microbiota (barrier effect). This allows overgrowth of pathogenic, opportunistic, or resistant microorganisms, potentially altering the ecological balance of the microbial community [4,5,6]. Despite the enormous amount of data on the effects of antimicrobials used in human medicine at therapeutic dose levels, which cause increased incidence of antimicrobial resistance, little is known regarding chronic or long-term exposure of residual veterinary drug levels in food, which could affect bacteria in the gastrointestinal tract and induce resistance [7,8,9]. The acceptable daily intake (ADI) is based on an array of toxicological, microbiological, and pharmacological data used in safety evaluations that take into account acute, chronic, and long-term exposure by ingestion of veterinary drug residues in food [7]. There are potential hazards of antimicrobial drug residues in food that include adverse effects on human intestinal microbiota composition, antimicrobial resistance, and intestinal permeability, leading to barrier disruption effects and selection of resistant intestinal bacteria [7,8,9].

Tetracycline is one of the most commonly used antimicrobials in human medicine and it is also used as a veterinary drug in food animals. Therapeutic levels of tetracycline used in human health can disrupt the balance of intestinal microbiota, develop resistant strains, and alter metabolic activity, which allows the overgrowth of pathogenic, opportunistic, or resistant microorganisms [10,11,12,13]. Several in vitro and in vivo studies mimicking potential human exposure to tetracycline were conducted to understand the effect of its residual levels on human intestinal microbiota [13,14,15,16,17,18,19]. Different microbiological endpoints were measured to detect changes in the microbiological community as the result of tetracycline exposure. Hirsh et al. [17] showed that an oral dose of 50 mg tetracycline/day did not change the number of *Escherichia coli* cells in the gut. Perrin-Guyomard et al. [18] estimated that the no-observed-effect level (NOEL) for tetracycline on intestinal microbiota was less than 1 µg/mL (equivalent to 0.125 mg/kg body weight (bw)/day based on a 40 g mouse water intake of 5 mL/day). Carman et al. [14] reported that 15 mg/60 kg equivalent bw/day (test concentrations of 1.5 µg/mL) of tetracycline showed no effect on microbiological endpoints, including total microbial number, short-chain fatty acids (SCFA) concentration, bile acids conversion, sulfate reduction, bacterial enzyme activity, or colonization resistance to *Clostridium difficile*. These values are approximately 10 to 30 times higher than the ADI set by the Joint Food and Agriculture Organization (FAO)/World Health Organization (WHO) Expert Committee on Food Additives (JECFA) (1.8 mg/60 kg bw/day, equivalent to 30 μg/kg bw/day) and U.S. FDA (1.5 mg/60 kg bw/day equivalent to 25 μg/kg bw/day) [20]. In another study on the effect of chlortetracycline intake in humans, Corpet [16] showed that 0.5 µg/mL chlortetracycline in water increased the number of resistant *E. coli* cells. The European Medicines Agency (EMA) determined the ADI value for tetracycline to be 0.18 mg/60 kg bw/day, equivalent to 3 μg/kg bw/day, based on a measured resistance endpoint [20,21]. Various experimental test systems and approaches were used to assess the safety of veterinary drug residues for human consumption. However, as mentioned above, the no-observed-adverse-effect concentrations (NOAEC) differed depending on the in vitro and the in vivo model test systems used as well as the interpretation of data derived from microbiological endpoints in the studies evaluated by reviewers [15,20]. Various international scientific committees and national regulatory agencies recognized the need for monitoring intestinal bacteria composition, changes of target microorganisms within the predominant microbiota, changes in levels of volatile short-chain fatty acids, bacterial hydrolytic and reductive enzymes, emergence of resistance, or barrier disruption endpoints and drug bioavailability in the gastrointestinal tract to establish acceptable antimicrobial residue levels in food [13,14,15,16,17,18,19,20]. In this context, a systematic methodological evaluation of current approaches is essential to address this methodological challenge to measure the effect of chronic antimicrobial agent exposure on human intestinal microbiota. The aim of this study was to evaluate current approaches, including traditional cultures and molecular analysis techniques, to measure the effects of tetracycline exposure on human intestinal microbiota using a continuous flow bioreactor model.

## 2. Results

**Figure 1:**
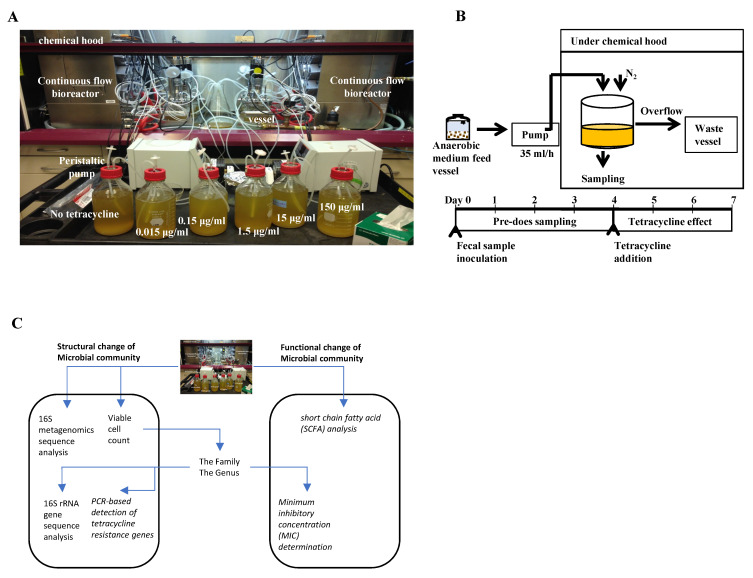
A laboratory continuous flow bioreactor (six reactors with 0, 0.015, 0.15, 1.5, 15, and 150 µg/mL tetracycline) (**A**); a schematic diagram and timeline (**B**); overview of measuring effects of tetracycline on human intestinal microbiota (**C**).

An overview of the type of outcome results expected to determine the impact of tetracycline exposure on the human intestinal microbiota in a continuous flow bioreactor model is shown in Figure 1. These results consist of structural and functional changes at genus, family, and microbial community levels. In order to assess the impact of NOAECs during chronic tetracycline exposure, data obtained on day 7 from controls (i.e., no tetracycline) and five tetracycline-treated bioreactor culture vessels were used.

After inoculation, the bioreactor cultures reached a steady state in approximately 3 days, which was confirmed by measuring counts of total anaerobic bacteria, pH, and concentration of SCFA in all bioreactor culture vessels. Furthermore, denaturing gradient gel electrophoresis (DGGE) was performed with a D Gene System (Bio-Rad Laboratories Inc., Hercules, CA, USA). The pH in all bioreactor culture vessels remained between 6.47–7.04 during pre-dose sampling. During this time, the pH of individual bioreactor culture vessels remained steady. Changes in the composition of a complex mixture of microorganisms were monitored daily by the SCFA profile and total anaerobic bacterial counts for 3 days before dosing with tetracycline (Figure S1A, Supplementary Material). During the pre-dose phase, the most prevalent fatty acids were acetate, propionate, and butyrate. The fatty acid that was detected in the lowest amounts was isobutyrate. The concentration of individual fatty acids showed some changes over time, but the total fatty acid content was moderately steady overall. Acetate was the most prevalent, representing 52–61% of the total SCFA. Propionate was found to be the second most prevalent, with 18–30% of the total. Other fatty acids included butyrate (18–22% of the total) and isobutyrate (1–10% of the total). Samples from the six test bioreactor culture vessels were cultured on Brain Heart Infusion Agar (BHI) and CDC Anaerobic Blood Agar (CDC) (Anaerobe Systems, Morgan Hill, CA, USA) plates and incubated under anaerobic conditions to monitor the total number of anaerobic bacteria cells as described below (Figure S1B,C, Supplementary Material). In the pre-dose phase, total anaerobic bacteria were present at concentrations ranging from 0.9 × 10^6^ to 13.6 × 10^6^ CFU/mL in all six bioreactor culture vessels. There were no significant changes in the total cell number of anaerobic bacteria in any of the six bioreactor culture vessels.

### 2.1. Structural Change of the Microbial Community

#### 2.1.1. Total Counts of Anaerobic Bacteria

**Figure 2:**
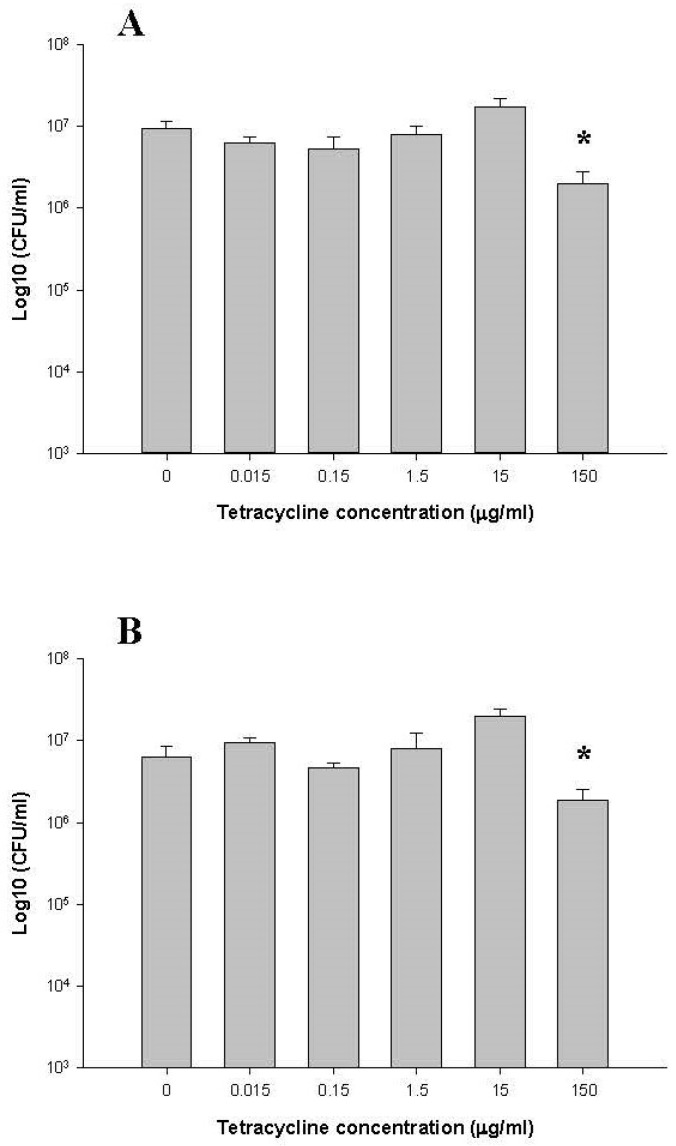
Comparison of total viable counts on BHI (**A**) and CDC (**B**) in controls (no treatment) and tetracycline-treated samples after 7 days. * indicates statistically significant differences from control (*p* < 0.05).

The number of anaerobic bacteria present in the bioreactors was monitored throughout the experiment by plating on BHI and CDC media. Figure 2 shows CFU/mL for each of the six bioreactor culture vessels. In the pre-dose phase (day 1–day 3), the numbers of total anaerobic bacteria on BHI and CDC media were present at 1.9 × 10^7^ ± 0.3 × 10^7^ and 1.7 × 10^7^ ± 0.5 × 10^7^ CFU/mL in all six bioreactor culture vessels. On day 7, three days after the addition of tetracycline, there was a 10-fold decrease (2 × 10^6^ ± 0.8 × 10^6^ and 1.8 × 10^6^ ± 0.7 × 10^6^ CFU/mL) in bacterial CFU in the 150 µg/mL tetracycline-dose bioreactor culture vessel (*p* < 0.05) (Figure 2). However, no changes in the number of total anaerobic bacteria cells were observed for the other tetracycline doses (i.e., 0, 0.015, 0.15, 1.5, and 15 µg/mL tetracycline) relative to the non-tetracycline dose.

To monitor the predominant bacterial species by culturing on selective agars, we chose selective culture media for the propagation of *Bifidobacterium* spp., *Fusobacterium* spp., *E. coli*, *Enterococcus faecalis*, *Eikenella corrodens*, and *Lactobacillus* spp. The number of anaerobic bacteria on Fusobacterium Selective Agar (FSA), CDC, and Lactobacillus-MRS Agar (LMRS) media during the pre-dose period varied from 3.5 × 10^3^ ± 0.1 × 10^3^ to 3.6 × 10^6^ ± 2.4 × 10^6^ CFU/mL. On day 7, no changes were observed on FSA media (2.3 × 10^6^ ± 1.4 × 10^6^ CFU/mL) in the 150 µg/mL tetracycline-dosed fecal suspension culture (Figure S2A, Supplementary Material). The recovery of *Lactobacillus, Eikenella corrodens* (betaproteobacteria), and *Bifidobacterium* on LMRS, Clindamycin Blood Agar (CBA), and Bifidobacterium Selective Agar (BIFIDO) was lower in the 150 µg/mL tetracycline dosed fecal suspension culture on day 7 (*p* < 0.05) (Figure S2B–D, Supplementary Material). On day 7, there were no observed changes of cell numbers on FSA media for fecal suspension cultures at other tetracycline doses relative to the non-tetracycline dose (Figure S2A, Supplementary Material).

#### 2.1.2. 16S Metagenomic Sequencing Analysis

**Figure 3:**
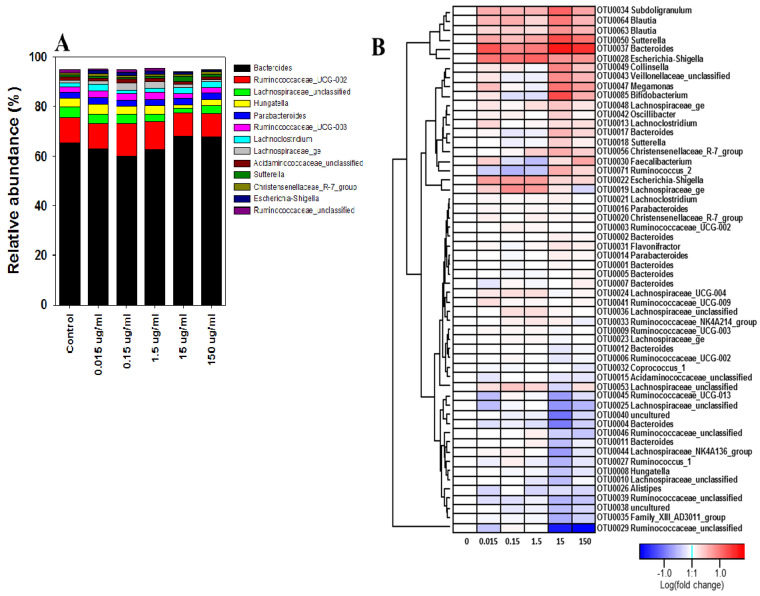
Bacterial communities change at the genus (**A**) and OTU levels (**B**) in controls (no treatment) and tetracycline-treated samples after 7 days. Heatmap (**B**) shows fold change of OTUs with >0.1% of relative abundance at control, and OTUs number/taxonomic group based on Mothur’s classification are represented for each row.

Illumina-based 16S rRNA gene amplicon sequencing was applied to bioreactor culture vessel samples to determine a tetracycline concentration that could impact the human intestinal microbial community structure. To assess the impact of tetracycline exposure, a total of 826,214 high-quality reads obtained from the 7 day tetracycline treated bioreactor culture isolates were used. For the control culture isolates from 7 day cultures, *Bacteroides* (65.53%), *Ruminococcaceae*_UCG-002 (10.15%), *Lachnospiraceae*_unclassified (4.07%), *Hungatella* (3.84%), *Parabacteroides* (2.27%), *Ruminococcaceae*_UCG-003 (2.34%), and *Lachnoclostridium* (1.38%) were detected at the genus level (Figure 3A). Evaluation of bacterial community changes at the genus level between controls and five tetracycline-treated culture samples taken between 4–7 days after treatment showed that bacterial communities were not significantly different (Figure 3A). Members of the genus *Bacteroides*, belonging to the phylum *Bacteroidetes*, increased at tetracycline treatments 15 and 150 µg/mL. However, operational taxonomic unit (OTU) level taxonomic profiling showed the effects of tetracycline exposure on the bacterial community changes in detail. Heatmap analysis for 57 OTUs with relative abundance of >0.1% for the control (non-treatment) showed that the pattern of change after tetracycline treatment varied for each OTU (Figure 3B) and could be classified into three groups. In particular, group I (3.23% of relative abundance), which consisted of minor OTUs with a relative abundance of <1.0% excluding OTU0013, tended to increase with increasing tetracycline dose. In group I, compared to the non-tetracycline treatment, *Subdoligranulum* OTU0034 and *Blautia* OTUs (0063 and 0064) increased for fecal suspension cultures exposed to all five tetracycline doses. The increase was at its highest for the 15 µg/mL tetracycline dose. *Sutterella* OTU0050 and *Bacteroides* OTU0037 also increased for all doses and tended to increase as the tetracycline dose increased. *Escherichia-Shigella* OTUs (0028 and 0022) also increased for all tetracycline doses, and the increase was at its highest for the 0.015–1.5 µg/mL tetracycline doses. *Collinsella* OTU0049, unclassified *Veillonellaceae* OTU0043, *Megamonas* OTU0047, *Bifidobacterium* OTU0085, *Lachnospiraceae_ge* OTU0048, *Oscillibacter* OTU0042, *Lachnoclostridium* OTU0013, *Bacteroides* OTU0017, *Sutterella* OTU0018, *Christensenellaceae* R-7 group OTU0056, *Faecalibacterium* OTU0030, and *Ruminococcus*_2 OTU0071 remained relatively constant for the 0.015–1.5 µg/mL doses but increased for 15 or 150 µg/mL tetracycline doses. *Lachnospiraceae_ge* OTU0019 increased for the 0.15 and the 1.5 µg/mL doses (Figure 3B). On the other hand, group II (71.06% of relative abundance), including 11 major OTUs (0001-3, 0005-7, 0009, 0012, and 0014-16) configuring relative abundance of 68.12%, tended to be relatively constant. Group III (21.29% of relative abundance) tended to be constant or decreased in the presence of tetracycline. *Ruminococcaceae* OTUs (0029, 0039, 0045, and 0046) and *Lachnospiraceae* OTUs (0025 and 0044) decreased for all tetracycline doses; the 15 µg/mL tetracycline dose showed the largest decrease.

#### 2.1.3. Abundance of the Family *Enterobacteriaceae* and the Genus *Escherichia*

**Figure 4:**
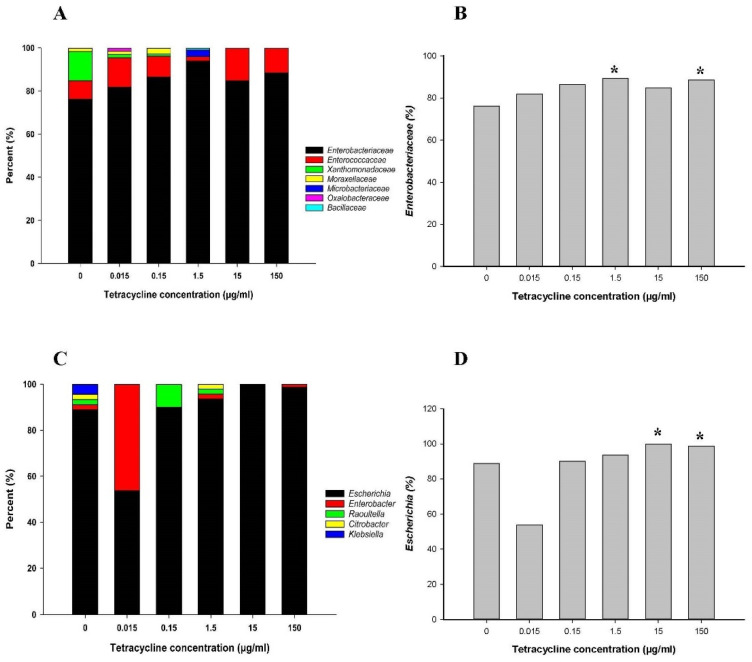
Changes in relative abundance of family (**A**), and genus (**B**), level and comparison of family *Enterobacteriaceae* (**C**), and genus *Escherichia* (**D**) in controls (no treatment) and tetracycline-treated samples after 7 days. * indicates statistically significant differences from control (*p* < 0.05).

From each treatment condition, approximately 100 bacterial colonies were randomly selected from BHI and CDC culture plates. A total of 532 isolates were obtained on day 7 from controls (i.e., 0 µg/mL tetracycline) along with five tetracycline-treated culture samples and were identified by a full length 16S rRNA gene sequence analysis. On day 7, the family level analysis from controls showed that the family *Enterobacteriaceae* of the phyla Proteobacteria (76.9%, 409/532) was predominant. Moreover, the families *Enterococcaceae* (8.5%) and *Bacillaceae* (0.2%) of the phyla Firmicutes, the families *Xanthomonadaceae* (1.9%), *Moraxellaceae* (0.9%), and *Oxalobacteraceae* (0.2%) of the phyla Proteobacteria, and the family *Microbacteriaceae* (0.6%) of the phyla Actinobacteria were identified (Figure 4A). However, after treatment with the 15 µg/mL tetracycline dose, the families Enterobacteriaceae and *Enterococcaceae* were predominantly present. To evaluate bacterial abundance changes on the family level between control and tetracycline-treated cultures sampled on day 7, we chose the family of *Enterobacteriaceae* (409 isolates) (Figure 4B). The abundance of the family *Enterobacteriaceae* increased slightly with a decrease in the abundance of *Xanthomonadaceae* and *Enterococcaceae* as the tetracycline dose increased. After treatment with the 0.015 µg/mL tetracycline dose, the family *Enterobacteriaceae* count increased by 2-fold (*p* < 0.05) (Figure 4B).

Within the family Enterobacteriaceae identified by sequence analysis, the predominant genera were *Escherichia* (88.8%, 363/409) and *Enterobacter* (7.1%) (Figure 4C). Apart from these, *Raoultella* (2.9%), *Klebsiella* (0.5%), and *Citrobacter* (0.7%) were also identified as minor populations. Although the distribution of these genera varied for different groups prior to tetracycline exposure, only the genera *Escherichia* and *Enterobacter* were present after treatment with 15 and 150 µg/mL tetracycline. Prominent changes for the genus *Escherichia* are shown in Figure 4D. After administration of tetracycline doses, changes were not observed at the genus level.

#### 2.1.4. Comparison of Tetracycline Resistance Genes in the Family *Enterobacteriaceae* and the Genus *Escherichia*

A total of 409 family Enterobacteriaceae isolates were screened for tetracycline resistance genes. Four tet genes (B, D, Q, and W) were found as major tetracycline resistance genes in the family *Enterobacteriaceae*. Two tet genes (M and O) were amplified as minor, and three tet genes (A, C, and X) were not amplified. The tetB was the most abundant, followed by tetW > tetD > tetQ in the cultures prior to tetracycline exposure.

**Figure 5:**
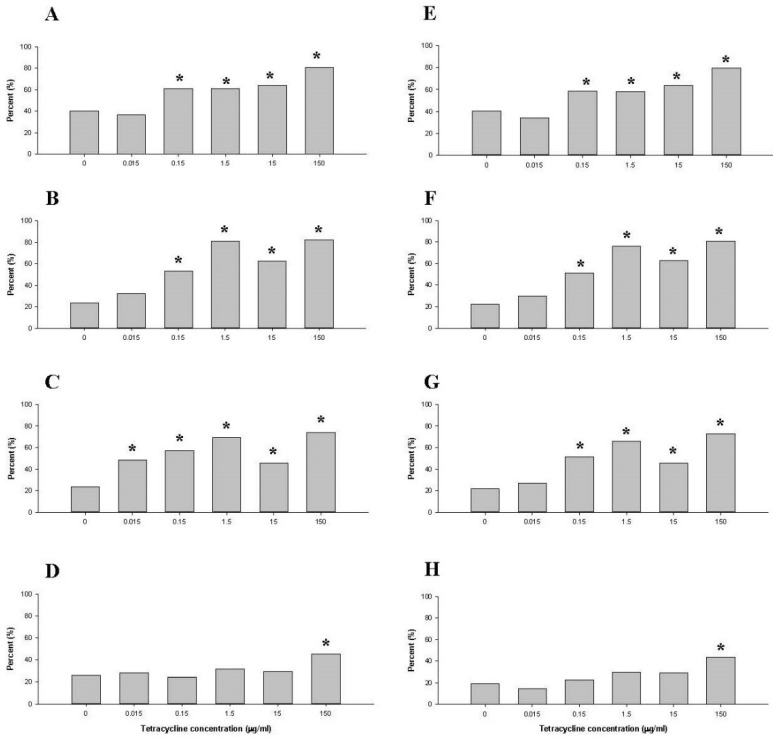
Comparison of percentage of four tetracycline resistance genes (*tetB*, *tetD*, *tetQ*, and *tetW*) as quantified by qRT-PCR in the family *Enterobacteriaceae* (**A**–**D**) and the genus *Escherichia* (**E**–**H**) in controls (no treatment) and tetracycline-treated samples after 7 days. *tetB*: (**A**,**E**), *tetD*: (**B**,**F**), *tetQ*: (**C**,**G**), *tetW*: (**D**,**E**). Quantification by qRT-PCR was performed in triplicate. Error bars indicate standard deviations. * indicates statistically significant differences from control (*p* < 0.05).

The percentages of tetB and tetD genes in the family *Enterobacteriaceae* were higher after treatment with the 0.15 µg/mL tetracycline dose than in control samples after 7 days of culture (*p* < 0.05) (Figure 5A,B). The percentage of tetQ was higher in the 0.015 µg/mL tetracycline dose than in the control sampled on day 7 (Figure 5C). The percentage of tetW fell within the 95% below and above the confidence interval, suggesting that even the highest dose of tetracycline (150 µg/mL) did not affect tetW (Figure 5D). After treatment with 0.15 µg/mL tetracycline, tetB, tetD, and tetQ of the genus *Escherichia* (Figure 5E–G) increased by 2-fold (*p* < 0.05). In contrast, tetW increased at tetracycline doses of 150 µg/mL (Figure 5H).

### 2.2. Functional Change of the Microbial Community

#### 2.2.1. Short Chain Fatty Acid (SCFA) Concentration Changes

**Figure 6:**
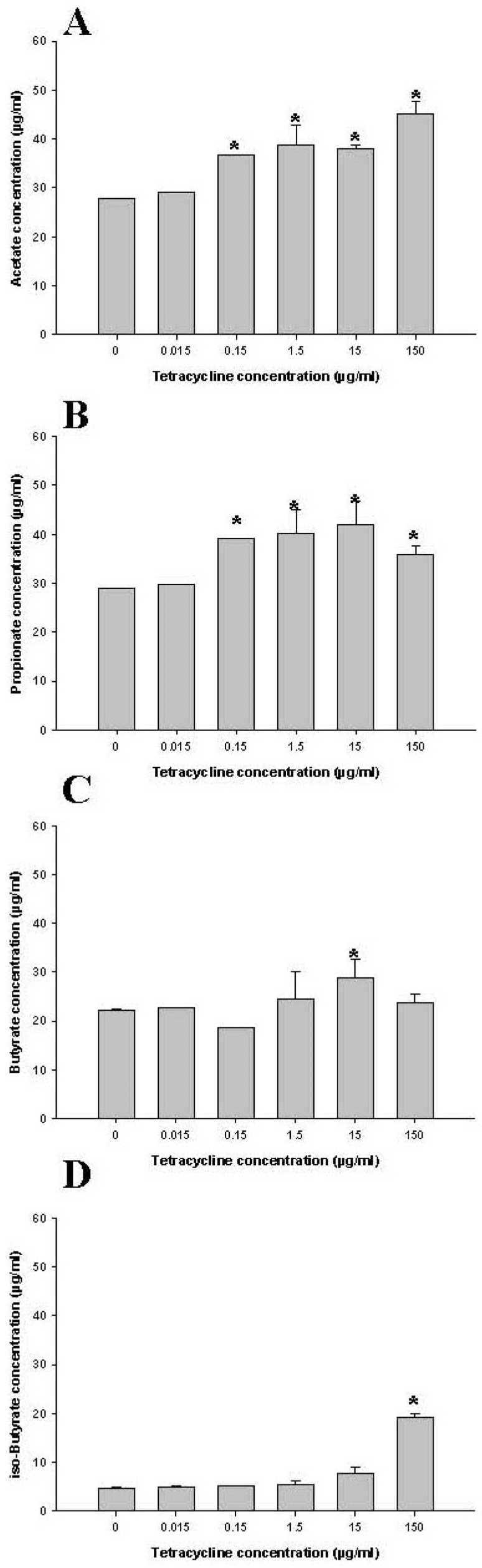
Comparison of acetate (**A**), propionate (**B**), butyrate (**C**), and isobutyrate (**D**) in controls (no treatment) and tetracycline-treated samples after 7 days. * indicates statistically significant differences from control (*p* < 0.05).

To assess any shifts in bacterial populations reflected by the metabolic parameter, changes in SCFA concentrations were determined (Figure 6). On day 7, SCFA were determined in controls (i.e., no tetracycline) as well as in human fecal samples treated with 0.015, 0.15, 1.5, 15, and 150 µg/mL tetracycline. Levels of acetate and propionate were higher in the cultures dosed with 0.15, 1.5, 15, and 150 µg/mL tetracycline than in control samples (*p* < 0.05), respectively (Figure 6A,B). The concentration of butyrate fell within the 95% below and above the confidence interval, suggesting that even the highest concentration of tetracycline (150 µg/mL) did not affect fatty acids (Figure 6C). The isobutyrate concentration was 10.3 ± 1.4 µg/mL on day 4 of 150 µg/mL tetracycline exposure and increased to 19.3 ± 0.7 µg/mL on day 7 (Figure 6D). Among the three dominant SCFAs, the concentration of acetate and propionate changed remarkably during tetracycline exposure, presenting a dose-dependent effect for those two fatty acids.

#### 2.2.2. Comparison of Minimum Inhibitory Concentration (MIC) for the Family *Enterobacteriaceae* and the Genus *Escherichia*

**Figure 7:**
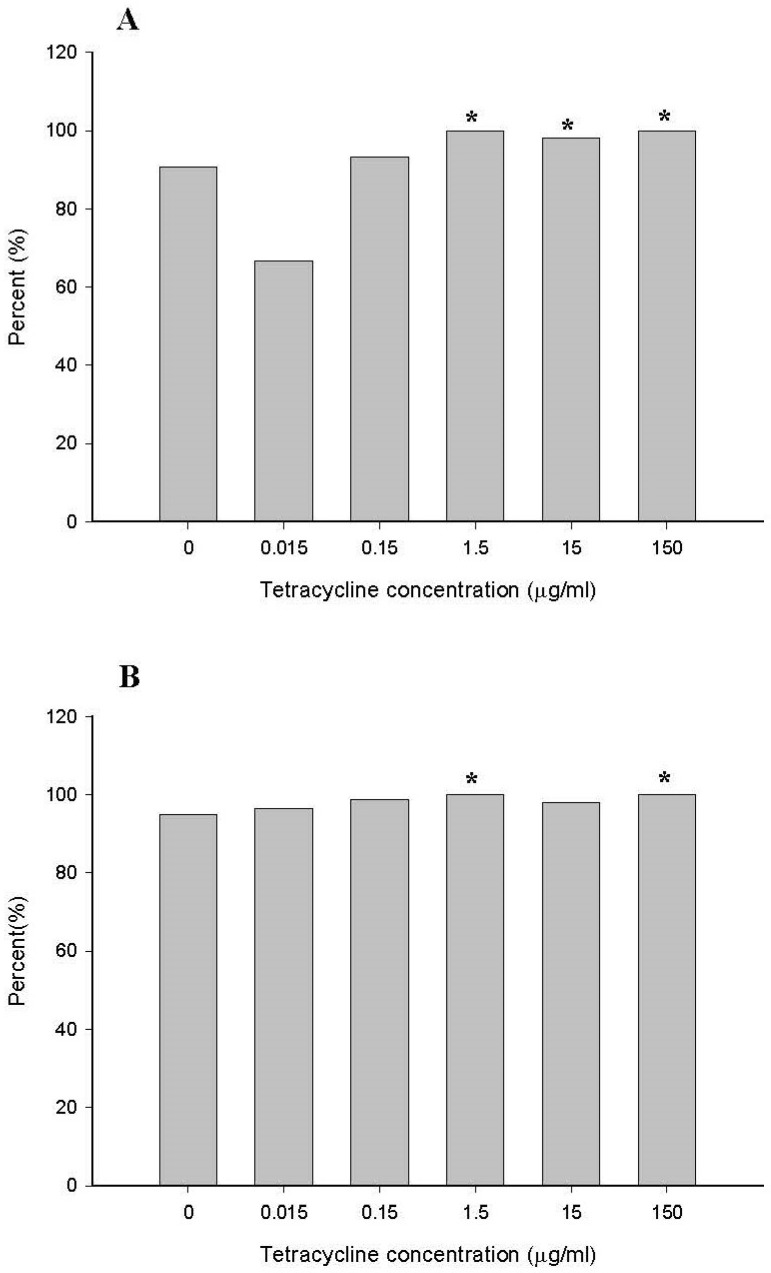
Comparison of percentage of tetracycline resistance (MIC > 16 µg/mL) of family *Enterobacteriaceae* (**A**) and genus *Escherichia* (**B**) in controls (no treatment) and tetracycline-treated samples after 7 days. * indicates statistically significant differences from control (*p* < 0.05).

*Enterobacteriaceae* was the predominant taxonomically diverse bacterial family. Members of this family can grow on BHI and CDC culture plates. Thus, for MIC determination, selected strains included the family *Enterobacteriaceae* (76.9%) and the genus *Escherichia* (88.8%) isolated from bioreactors according to the methods defined in the CLSI guidelines [22]. A total of 409 family *Enterobacteriaceae* isolates were subjected to drug susceptibility testing. To compare the MIC between controls to 0.015, 0.15, 1.5, 15, and 150 µg/mL tetracycline-treated human fecal samples, the tetracycline breakpoint (MIC ≥ 16 µg/mL) concentration was chosen [23] and calculated for the family *Enterobacteriaceae* (Figure 7A) and the genus *Escherichia* (Figure 7B). From the no-tetracycline-dose bioreactor on day 7, approximately 90% of the isolated bacteria belonging to the family *Enterobacteriaceae* exhibited high-level resistance to tetracycline (MIC ≥ 16 µg/mL). In 1.5, 15, and 150 µg/mL tetracycline-treated human fecal samples, 98% to 100% of the family *Enterobacteriaceae* showed a tetracycline breakpoint (MIC ≥ 16 µg/mL). The percentages of tetracycline breakpoints in the family *Enterobacteriaceae* were higher in the cultures does with 1.5, 15, and 150 µg/mL tetracycline than in control samples (*p* < 0.05) (Figure 7A). In the genus *Escherichia*, 95% from the non-tetracycline dose bioreactor on day 7 exhibited a high-level resistance to tetracycline. After treatment with 0.15 µg/mL tetracycline, the genus *Escherichia* showed slightly increased MIC levels of tetracycline breakpoints (*p* < 0.05) (Figure 7B).

## 3. Discussion

Tetracycline and other antimicrobial drug residues may be present in edible tissues and meat products from treated food-producing animals. These residues are considered to be potential hazards because they can affect human intestinal microbiota composition, antimicrobial resistance, and intestinal permeability, leading to barrier disruption effects and selection of resistant intestinal bacteria [7,8,9,13,15,19,20,24]. Microbiological endpoints that should be considered when establishing a microbiological acceptable daily intake (mADI) are the disruption of the colonization barrier and the increase of resistant bacterial populations [9]. Previously, researchers monitored intestinal bacteria composition changes within the predominant microbiota, changes in levels of volatile SCFAs, bacterial hydrolytic and reductive enzymes, emergence of resistance, barrier disruption endpoints, and drug bioavailability in the gastrointestinal tract to establish acceptable antimicrobial residue levels in food [7,14,15,16,17,18,19,25,26,27]. The goal of this study was to evaluate current approaches to measuring chronic exposure of tetracycline on human intestinal microbiota using a continuous flow bioreactor. Strengths and weaknesses of evaluating current approaches and methods in a continuous flow bioreactor are listed in Table 1.

While researchers routinely rely on culture-based approaches to study NOAECs [14,18,25,26,28,29,30], there is a need to include modern molecular techniques, pyrosequencing, and metagenomics to evaluate abundant microorganisms [19,31,32]. Unlike conventional microbiological culture methods, high-throughput pyrosequencing and metagenomics approaches for microbial community analysis led to a more detailed understanding of the complexity of human intestinal microbiota and community structure. Indeed, only an estimated 50% of the operational taxonomic units (OTUs) detected by 16S rRNA gene sequencing of fecal samples were isolated and characterized [33,34,35]. In this study, *Bacteroides* was 65.53% detected at the genus level by 16S rRNA metagenomics sequencing analysis, whereas the family *Enterobacteriaceae* (76.9%) and the genus *Escherichia* (88.8%) were predominant from BHI and CDC plates. These different results between metagenomics sequencing analysis and culture-based approaches can be explained by how the family *Enterobacteriaceae* would grow more quickly on BHI and CDC media and are easily isolated from human intestinal microbiota, although genus *Escherichia* accounts for approximately only 0.1% of the microbes inhabiting the average human intestinal microbiota [36]. In addition, molecular methods neglect minority populations at concentrations lower than approximately 10^5^ CFU/mL [37,38]. Among these neglected populations are bacteria detected on FSA, CDC, and LMRS media, which may be present in bioreactor cultures at low threshold concentrations. In this study using culture-based approaches, selective screening for *Bifidobacterium, Fusobacterium,* and *Lactobacillus* at 0.015, 0.15, 1.5, and 15 µg/mL tetracycline concentrations for four consecutive days indicated that these low drug levels did not disrupt the initial balance of human intestinal microbiota (data not shown). Administration of a high dose of tetracycline (150 µg/mL) suppressed the growth of anaerobic bacteria in the bioreactor. Based on Illumina 16S amplicon sequencing results, the relative abundance changes at the genus level were constant between controls and tetracycline-treated samples. In our previous investigations based on 16S rRNA gene-targeting qRT-PCR approach, we consistently showed that total bacterial cells from controls to five tetracycline-treated culture samples were not different [19]. However, these results of a high dose of tetracycline did not overlap between the metagenomics sequencing analysis and the culture-based approach. The most logical reason for this could be that these culture-based methods, including enrichment using BHI or CDC, may be limited by their high specificity along with low sensitivity. Our results further emphasize that detailed experimental studies/evidence for the possibility of resistance on BIFIDO, FSA, and LMRS media need to be evaluated through further investigations. Furthermore, these culture-independent DNA sequencing methods cannot distinguish live from dead cells, which can lead to overestimating viable bacterial cells at a high tetracycline dose [39]. Overall, a variety of factors can disturb the determination of indigenous intestinal microbiota and may be limited to evaluation of NOAECs. Although microbial community perturbations were observed with increasing tetracycline concentrations, NOAECs were difficult to evaluate by bacterial community changes at or above the genus level.

SCFAs are the principal products of carbohydrate and protein fermentation and are some of the most important physiologic processes mediated by intestinal microbiota [40,41,42]. Different groups of bacteria exhibit distinct patterns of fermentation product formation according to environmental conditions, including pH, partial hydrogen pressure, and available substrates [43]. If the level of an antibiotic introduced into a bioreactor is high enough to affect a bacterial population, this can manifest itself by selective cell death and/or a decrease in the total population of microbial cells within the bioreactor culture. In our results, acetate, propionate, and butyrate were found to be dominant SCFAs in the control and in tetracycline-dosed human fecal suspension samples. These results were consistent with earlier SCFA studies in that the major fermentation products in the gut of healthy adults were acetate, propionate, and butyrate (typically in a 3:1:1 ratio) [14,25,29,30,43]. On day 4, after the addition of tetracycline into bioreactor culture vessels, the concentrations of acetate decreased, with propionate and butyrate being the least affected. Acetate is produced by bifidobacterial, lactobacilli, acetogenic bacteria, and by most enteric bacteria as a fermentation product [43,44,45]. Butyrate and propionate are produced by members of the *Bacteroides* phylum and the *Clostridium* clusters XIVa and IV [44,45]. We observed no changes in percentage of *Enterobacteriaceae* (409 isolates) or *E. coli* (363 isolates) as the tetracycline concentration increased. Although the specific roles of microbiota in the bioreactor cultures remain unknown, the study data indicate that acetate and propionate levels in the bioreactor culture vessels became higher with increasing tetracycline concentration, whereas butyrate levels remained constant. These variable results could be due to changes in unculturable or slow growing bacterial populations.

The selection of resistant bacteria as an endpoint is of high public health concern due to observed resistance to the same classes of tetracyclines used in human and veterinary medicine [46,47]. Aerobic and anaerobic bacteria are susceptible to tetracycline, i.e., more than 50% of the strains were inhibited below the breakpoint value of 16 µg/mL [23]. Typical MIC values for tetracycline against organisms such as *Bifidobacterium* spp., *Fusobacterium* spp., and *Lactobacillus* spp. were shown to be in the range of 0.116 to >256 µg/mL [46,47,48]. In our study, there were 532 bacteria isolated from BHI and CDC plates, and they showed an average MIC of 85 µg/mL. In the non-tetracycline dose bioreactor culture samples, most (90%) of the family *Enterobacteriaceae* showed high levels of resistance to tetracycline (>16 µg/mL). This may be due to the inherent resistance to tetracycline in the family *Enterobacteriaceae*. The efflux genes (*tetA*, *B*, *C*, *D*, and *E*) are frequently detected in the family *Enterobacteriaceae* [49,50,51]. In this study, six *tet* genes (*B*, *D*, *M*, *O*, *Q*, and *W*) were found, and three *tet* genes (*A, C,* and *X*) were not amplified. Similarly, *Enterobacteriaceae* isolated from dairy farm soil exhibited a higher frequency of seven *tet* genes (*A*, *B*, *G*, *M*, *O*, *S*, and *W*), whereas other *tet* genes (*C*, *D*, *E*, *K*, *L*, *Q*, and *T*) were not detected [52]. Despite this, tetracycline administration in our study did not lead to an increased percentage of the family *Enterobacteriaceae* or the genus *Escherichia*; percentages of *tetB* and *tetD* genes in the family *Enterobacteriaceae* and the genus *Escherichia* were consistent with increased tetracycline concentrations of 0.15 µg/mL and above. Resistance detected here appeared to be related to tetracycline concentration and to the presence of multiple tetracycline resistance genes in a resistant bacterial population. In this study, it is possible that selection pressures provided by the elevated levels of tetracycline could have led to the acquisition of more than one tetracycline gene in a given strain due to their prevalence in the tetracycline-dose bioreactor cultures. Some previous studies suggest that multiple *tet* genes can be present at over 20% frequency within Gram-negative bacteria in some ecosystems [52,53,54]. Here, selection pressure created by the presence of tetracycline possibly increased transfer frequencies of mobile genetic elements, which can carry tetracycline resistance genes. In general, the results of tetracycline resistance genes detected in this investigation were very similar to those obtained from in vitro fecal slurries reported by Jung et al. [19]. For reasons not yet understood, the fecal donor in this study was populated with these resistant strains despite the absence of recent antibiotic use. To obtain useful data, target bacteria in the fecal samples should be pre-screened for resistance to the antibiotic to be tested. This would increase the likelihood of obtaining meaningful results on changes in antibiotic resistance.

The continuous flow bioreactor system is one of the in vitro test methodologies recommended in the VICH GL 36(R2) to determine NOAECs for functional endpoints, including hydrolytic and reductive enzyme reactions, gas production, volatile/nonvolatile fatty acid formation, bacterial interactions, colonization barrier disruption, and resistance emergence [14,15,20,26,28,55]. The advantages of using a bioreactor are that it provides (1) an ecosystem which mimics microbiota interactions in the human intestine and (2) measurable microbiological endpoints that can be compared with those measured in humans [15,20]. However, operation of this complex culture system requires technical expertise to achieve microbial populations at levels similar to those found in vivo [15,20]. In this study, during the continuous flow bioreactors experiment, creating a steady state environment that maintains constant biomass and nutrient concentration within six bioreactor culture vessels constituted a particular challenge. Generally, total anaerobic bacterial counts and total SCFA concentration became steady within 3 days. Moreover, the denaturing gradient gel electrophoresis (DGGE) band patterns (i.e., the V3 region of the 16S rRNA gene) of the bacterial community in pre-dose samples from six bioreactor culture vessels on the initial day (day 0) were similar to those on day 3 (data not shown). Achievement of a steady state is a major requirement when using such bioreactor systems to assess the impact of low tetracycline concentrations on human intestinal microbiota.

Previous investigations showed that the NOAECs differed depending on the experimental test systems and approaches used [14,15,16,17,18]. There is a need to design research protocols that are relevant and reproducible to determine the magnitude of change that would occur in commensal and resistant populations after their exposure to antimicrobial drug residues found in food [7,9]. This pilot study needs to be repeated with fecal samples from more human subjects, taking into consideration interindividual variability of the intestinal microbiota and background tetracycline resistance levels [19,31]. Using traditional culture techniques to monitor bacteria in bioreactors is laborious and time-consuming. Furthermore, these low-resolution techniques only allow detection of substantial changes in bacterial community structure. However, the incorporation of molecular methods to monitor population shifts could reduce assay time. These high-resolution methods also allow detection of minute changes in bacterial community structure and function [19]. This is beneficial both in sensitivity and specificity when compared with traditional techniques to monitor changes in intestinal bacteria composition. Further research into improving novel tools would include analytical chemistry approaches, high-throughput pyrosequencing, metagenomics, metatranscriptomics, metaproteomics, and metabolomics studies.

## 4. Materials and Methods

### 4.1. Human Fecal Samples

Fecal samples were collected from a healthy male subject who had not received antibiotics within the past six months. The use of human fecal samples was approved by the FDA Research Human Subjects Committee (Approval # 14-061T). Fresh fecal samples from this volunteer were collected by direct passage into the Commode specimen collection system (Fisher Scientific, Pittsburgh, PA, USA). Samples were transferred to an anaerobic chamber hood for making homogeneous suspensions by distributing them into separate vessels. These samples were then pooled and uniformly supplemented with a pre-reduced anaerobically sterilized low-carbohydrate medium (LCM) [56] to prepare 3% (*w*/*v*) suspensions [19].

### 4.2. Establishment of Continuous Flow Bioreactor Cultures

Fecal samples were independently diluted with LCM to make a 3% fecal slurry (*w*/*v*). A total of 350 mL of this slurry was aliquoted to each of six culture vessels within a bioreactor (Biostat Qplus, Sartorius AG, Göttingen, Germany). The bioreactor consisted of six 500 mL culture vessels, a feed bottle, and a waste bottle (Figure 1A). Vessels in the bioreactor were anaerobically maintained at 37 °C under continuous agitation (at 50 rpm) for 7 days. Sterile culture medium was pumped from the feed vessel into the culture vessel at a rate of 10.8 mL/h (equivalent to a dilution rate of 0.031/h; 259 mL turnover per day, retention time: 32.43 h). To maintain a constant culture vessel volume, spent culture medium was removed from the vessel at a rate identical to the inflow.

On day 4, each tetracycline dose (0 (control), 0.015, 0.15, 1.5, 15, or 150 µg/mL) was dissolved and placed into an LCM feed bottle, which was then pumped into a designated bioreactor culture vessel (Figure 1B). These doses corresponded to ADI values of 0, 2.5, 25, 250, 2500, or 25,000 µg/kg bw/day, assuming that approximately 100 µg of tetracycline can be present in 1 g of feces [14]. Tetracycline concentrations were intended to simulate exposure levels at equivalent, below, and above the corresponding codified ADI for the United States guideline of 25 µg/kg bw/day [57]. Before and after addition of tetracycline to the bioreactor culture vessels, a 10 mL sample was removed from each vessel daily on culture days 1–7 for microbiological endpoint measurements.

### 4.3. Tetracycline Analysis by HPLC and LC-MS/MS

Tetracycline was detected in an aqueous phase by HPLC and LC-MS/MS as previously described [24]. A 1 mL sample of dosed fecal suspension culture was collected from each bioreactor culture vessel between days 4 and 7, centrifuged at 10,000× *g* for 20 min, and filtered (0.2 µm, 25 mm, Millipore, Billerica, MA, USA). The supernatants were evaluated for tetracycline concentration by HPLC (15 and 150 µg/mL) and LC/MS (0.015, 0.15 and 1.5 µg/mL) (Table S1, Supplementary Material) [24].

### 4.4. Structural Assessment of Microbiological Endpoints

#### 4.4.1. Viable Bacterial Counting at the Microbial Community-Level

A 1 mL sample from each of the six culture vessels was used to prepare a 10-fold dilution series in anaerobic maximum recovery diluent (MRD; LabM IDG, Bury, UK) [56]. Ten microliters of each serial dilution were placed onto commercially prepared BHI, BIFIDO, FSA, CBA, LMRS, and CDC (Anaerobe Systems, Morgan Hill, CA, USA). Total anaerobic counts were made on BHI and CDC media. Different culture media were used for selective culture of different bacteria groups: *Bifidobacterium* spp. (BIFIDO), *Fusobacterium* spp. (FSA), *E. coli*, *Enterococcus faecalis*, *Eikenella corrodens* (CBA), and *Lactobacillus* spp. (LMRS). Inoculated plates were incubated in an anaerobic glove box (Coy Laboratory Products, Grass Lake, MI, USA) for 2 days at 37 °C.

#### 4.4.2. 16S rRNA Metagenomics Sequencing Analysis at the Microbial Community Level

One milliliter of each fecal suspension culture sample from each vessel daily on culture days 1–7 was centrifuged at 10,000× *g* for 20 min, and the pellets were stored at −80 °C. Total DNA was extracted from each pellet of the triplicate samples using a DNeasy PowerSoil Kit according to the manufacturer’s instructions (Qiagen, Germantown, MD, USA). Hypervariable regions (V3–V4) of bacterial 16S rRNA genes were PCR-amplified based on the Illumina 16S metagenomics sequencing library preparation guide [58]. Two PCR reactions were completed on the template DNA. Initially, the oligonucleotide primers consisted of Illumina adapter overhang sequences. V3–V4 specific sequences were used to start the first PCR reaction. The Amplicon PCR reaction mixture (25 μL) contained a DNA template (5 ng), 5 μL of forward primer (1 μM), 5 μL of reverse primer (1 μM), 12.5 μL of 2× KAPA HiFi HotStart ReadyMix (Roche Sequencing Store, Wilmington, MA, USA), and PCR-grade water. PCR amplification was carried out in a T100^™^ thermal cycler (Bio-Rad Laboratories Inc., Hercules, CA, USA) as follows: initial denaturation at 95 °C for 3 min, followed by 25 cycles of 95 °C for 30 s, 55 °C for 30 s, and 72 °C for 30 s, then 72 °C for 5 min and held at 4 °C. PCR products were purified using a QIAquick PCR purification kit (Qiagen, Germantown, MD, USA), and the purified PCR product (5 μL) was used for a second PCR reaction to add the Illumina sequencing adapters and dual-index barcodes. Each PCR reaction contained 5 μL of Illumina Nextera XT index 1 primer (N7xx), 5 μL of Nextera XT index 2 primer (S5xx), 25 μL of 2× Kapa HiFi HotStart ReadyMix, and 10 μL of PCR grade water, and PCR amplification was completed as follows: initial denaturation at 95 °C for 3 min, followed by 8 cycles of 95 °C for 30 s, 55 °C for 30 s, and 72 °C for 30 s, then 72 °C for 5 min and held at 4 °C. PCR products were purified again using a QIAquick PCR purification kit. The pooled final DNA library was sequenced on Illumina MiSeq platform using the paired end (2 × 300 bp) option at Axeq Technologies (Macrogen Inc., Rockville, MD, USA).

Sequencing reads were processed using the Mothur MiSeq SOP [59]. The dataset was demultiplexed based on unique barcodes. Barcodes, adapter, and universal primer sequences were then removed. After assembling paired end reads into contigs, the latter were removed if they were <300 bp, >700 bp, or contained any ambiguous base calls. Resulting contig data were aligned to a SILVA v.132 reference alignment curated to the V3–V4 region and pre-clustered, allowing for two nucleotide differences between sequences. Chimeric reads were removed using the Mothur VSEARCH algorithm [60]. The high-quality reads were classified into taxonomic lineage using the *k*-nearest neighbor (*k*-NN) algorithm. “Undesirable” sequences belonging to chloroplast, mitochondria, unknown archaea, and eukaryote lineages were removed. Moreover, the high-quality reads were clustered into OTUs at 97% sequence similarity. The OTUs were then classified.

A heat map analysis was performed using the heatmap.2 function found in the gplots package [61] of the R program to analyze the abundance change at the OTU level by tetracycline treatment. Illumina sequencing raw data were deposited in the NCBI Sequence Read Archive (SRA) with accession no. PRJNA384806.

#### 4.4.3. Isolation and 16S rRNA Sequencing Analysis at the Family Level

From each treatment condition, approximately 100 bacterial colonies were isolated from BHI and CDC plates that were inoculated on day 7. These isolates were maintained at 4 °C on Trypticase Soy Agar (TSA) supplemented with 5% sheep blood (Blood Agar; BA). For long-term storage in 96-well plates, cultures in the exponential growth phase were stored at −80 °C after the addition of glycerol (10%, vol/vol).

These frozen isolates were thawed, grown on TSA medium, and evaluated for taxonomic identification. Colonies were transferred into Eppendorf tubes containing 0.1 mL of InstaGene Matrix (Bio-Rad Laboratories Inc., Hercules, CA, USA), boiled for 10 min in a water bath, and then centrifuged at 10,000× *g* for 5 min. Five microliters of the supernatant were used for PCR. Universal primers 27F and 1492R and multiple internal primers [62] were used to amplify the 16S rRNA gene of isolates using the conditions described by Jung et al. [19]. PCR products were purified using a QIAquick PCR purification kit (Qiagen, Germantown, MD, USA), and sequences were determined by Macrogen Inc (Rockville, MD, USA). Using an in-house python script for performing a BLAST search locally in the NCBI 16S rRNA database by means of data parsing, the amplified 16S rRNA gene sequences were taxonomically annotated at the species level.

#### 4.4.4. PCR-Based Detection of Tetracycline Resistance Genes at the Family Level

The presence of tetracycline resistance genes was determined by quantitative real-time PCR (qRT-PCR). We assayed 9 *tet* genes (four for efflux pump: *tetA*, *B*, *C*, and *D*; four for ribosomal protection: *tetM*, *O*, *W*, and *Q*; and one gene for antibiotic inactivation: *tetX*) using primer sets as previously described [19,63].

### 4.5. Functional Assessment of Microbiological Endpoints

#### 4.5.1. SCFA Analysis at the Microbial Community-Level

One milliliter samples of dosed fecal suspension cultures were centrifuged at 10,000× *g* for 20 min and filtered (0.2 µm, 25 mm, Millipore, Billerica, MA, USA) as described above. The supernatants were evaluated for SCFA by HPLC with an Aminex^®^ HPX-87H column (300 × 7.8 mm; Bio-Rad Laboratories Inc., Hercules, CA, USA) with UV detection at 210 nm as previously described [31]. A mobile phase of 0.2 N H_2_SO_4_ at a flow rate of 0.6 mL/min was used.

#### 4.5.2. MIC Determination at the Family Level

Bacterial strains isolated from dosed bioreactor culture vessels were subjected to MIC determination according to the methods defined in the Clinical and Laboratory Standards Institute guidelines (CLSI) [22]. The MIC of tetracycline for isolates was determined in a 96-well microtiter plate. Serial dilutions of tetracycline in concentrations ranging from 0.25 to 256 µg/mL were prepared in 200 µl of Mueller-Hinton broth (MHB, LabM, Farmingdale, NY, USA). The wells were inoculated with 2 × 10^5^ CFU/mL, and the plates were incubated for 24 h at 37 °C. The MIC of tetracycline, in which no bacterial growth was observed, was then determined. *E. coli* ATCC25922 was used as quality control for MIC determination.

### 4.6. Statistical Analyses

Statistical analyses of viable bacterial counting and SCFA analysis at the microbial community level were performed using a one-way analysis of variance (ANOVA) and post-hoc Dunn’s test using SigmaPlot vs. 13.0 software, with a *p* value of <0.05 being considered significant. To compare the control and the tetracycline treatment (i.e., control vs. each tetracycline treatment before and after receiving a particular tetracycline treatment) for 16S rRNA sequencing analysis, PCR-based detection of tetracycline resistance genes, and MIC determination at the family level, statistical analysis was performed between groups using two-tailed Fisher exact probability test using GraphPad web-based software (https://www.graphpad.com/quickcalcs/contingency1/) (accessed on 15 June 2021).

## 5. Conclusions

Chronic exposure to different tetracycline concentrations via consumption of various animal products is associated with dose-dependent changes in microbial populations obtained from human fecal sample-seeded bioreactor cultures. The endpoints evaluated in this investigation were based on structural and functional changes in the microbial community from an in vitro continuous culture bioreactor model system. Results from one human donor fecal suspension inoculated into an in vitro continuous flow bioreactor model with exposure to tetracycline at high concentrations show that higher concentrations of the antibiotic can affect human intestinal microbiota. According to results from the traditional culture and the molecular analysis techniques used in this study, NOAECs were shown to vary. Thus, continuous flow bioreactor model systems combined with meta-omics approaches can be used as effective tools to assess the impact of chronic drug exposure to human intestinal microbiota.

## Figures and Tables

**Table 1:** Summary table of evaluating methods used in a continuous flow bioreactor.

Methods		Strengths	Weaknesses	References
Structural change	Viable cell count	Easy and rapid screening Cost effective	Laborious and time-consuming process Low-resolution techniques Lack of sensitivity	Carman et al. [14] Corpet [16] Hirsh et al. [17] Perrin-Guyomard et al. [18]
	16S metagenomics sequence analysis PCR-based detection of tetracycline resistance genes 16S rRNA gene sequence analysis	Reduce assay time High-resolution techniques Sensitive and specific techniques Indigenous intestinal bacteria	Expensive equipment and trained staff Data interpretation Extensive sample preparation prior to analysis	Jung et al. [19] Kim et al. [32]
Functional change	Short chain fatty acid (SCFA) analysis	Accurate technique for quantitative analysis Does not require extraction steps Rapid for time course Indigenous intestinal bacteria	Expensive equipment and trained staff Data interpretation	Carman et al. [14] Perrin-Guyomard et al. [18]
	Minimum inhibitory concentration (MIC) determination	Rapid screening techniques Standard procedures Designed and optimized for the evaluation of pathogenic clinical isolates	Laborious and time-consuming process Not commensal intestinal bacteria Does not take into account the ecological interactions of bacteria in gastrointestinal tract	Carman et al. [14] Wagner et al. [13]

## Supplementary Materials

The following are available online at https://www.mdpi.com/article/10.3390/antibiotics10080886/s1, Figure S1: Plot of short chain fatty acid (SCFA) concentrations (A) and cell number on BHI (B) and CDC (C) for the six bioreactors over the course of the experiment. Figure S2: Comparison of *Fusobacterium*, *Lactobacillus, Eikenella corrodens* (betaproteobacteria), and *Bifidobacterium* viable counts on FSA (A), LMRS (B), CBA (C), and BIFIDO (D) in controls (no treatment) and tetracycline-treated samples after 7 days. * indicates statistically significant differences from control (*p* < 0.05). Table S1: Nominal levels of test compound added to the medium and measured by bioassay.
