# Supplementary material for: Impact of Chronic Tetracycline Exposure on Human Intestinal Microbiota in a Continuous Flow Bioreactor Model
Source: Antibiotics (Basel). 2021 Jul 21;10(8):886. doi: 10.3390/antibiotics10080886 (PMC8388752; doi:10.3390/antibiotics10080886)
Supplement: Supplementary file 1 [file antibiotics-10-00886-s001.zip › antibiotics-1282872-supplementary.pdf]

antibiotics

Supplementary Material

Impact of Chronic Tetracycline Exposure on Human Intestinal Microbiota in a Continuous Flow Bioreactor Model

Youngbeom Ahn [1,*,†], Ji Young Jung [1,†,‡], Ohgew Kweon [1], Brian T. Veach [2], Sangeeta Khare [1], Kuppan Gokulan [1], Silvia A. Piñeiro [3] and Carl E. Cerniglia [1]

[1] Division of Microbiology, National Center for Toxicological Research, U.S. Food and Drug Administration, Jefferson, AR 72079, USA; jyjung@nnibr.re.kr (J.Y.J.); oh-gew.kweon@fda.hhs.gov (O.K.); sangeeta.khare@fda.hhs.gov (S.K.); kuppan.gokulan@fda.hhs.gov (K.G.); carl.cerniglia@fda.hhs.gov (C.E.C.)

[2] Office of Regulatory Affairs, Arkansas Laboratory, U.S. Food and Drug Administration, Jefferson, AR 72079, USA; brian.veach@fda.hhs.gov

[3] Division of Human Food Safety, Center for Veterinary Medicine, U.S. Food and Drug Administration,
Rockville, MD 72079, USA; Silvia.Pineiro@fda.hhs.gov

* Correspondence: young.ahn@fda.hhs.gov; Tel.: +1-870-540-7084

† These authors contributed equally to this work

‡ Present address: Environmental Microbiology Research Team, Microbial Research Department, Nakdonggang National Institute of Biological Resources (NNIBR), 137, Donam 2-gil, Sangju-si, Gyeongsangbuk-do 37242, Republic of Korea

Table S1. Nominal levels of test compound added to the medium and measured by bioassay (mean level, *n* = 3).

Equivalent daily dose (mg/60 kg person/d)	Tetracycline (µg/ml)	
	Added	Recovered
0	none	not detected
0.15	0.015	0.015 ± 0.01
1.5[a]	0.15	0.17 ± 0.13
15	1.5	1.4 ± 0.72
150	15	16 ± 1.23
1500	150	154 ± 9.54

1.5[a]: the ADI was set by the U.S. FDA (FDA, 1996).

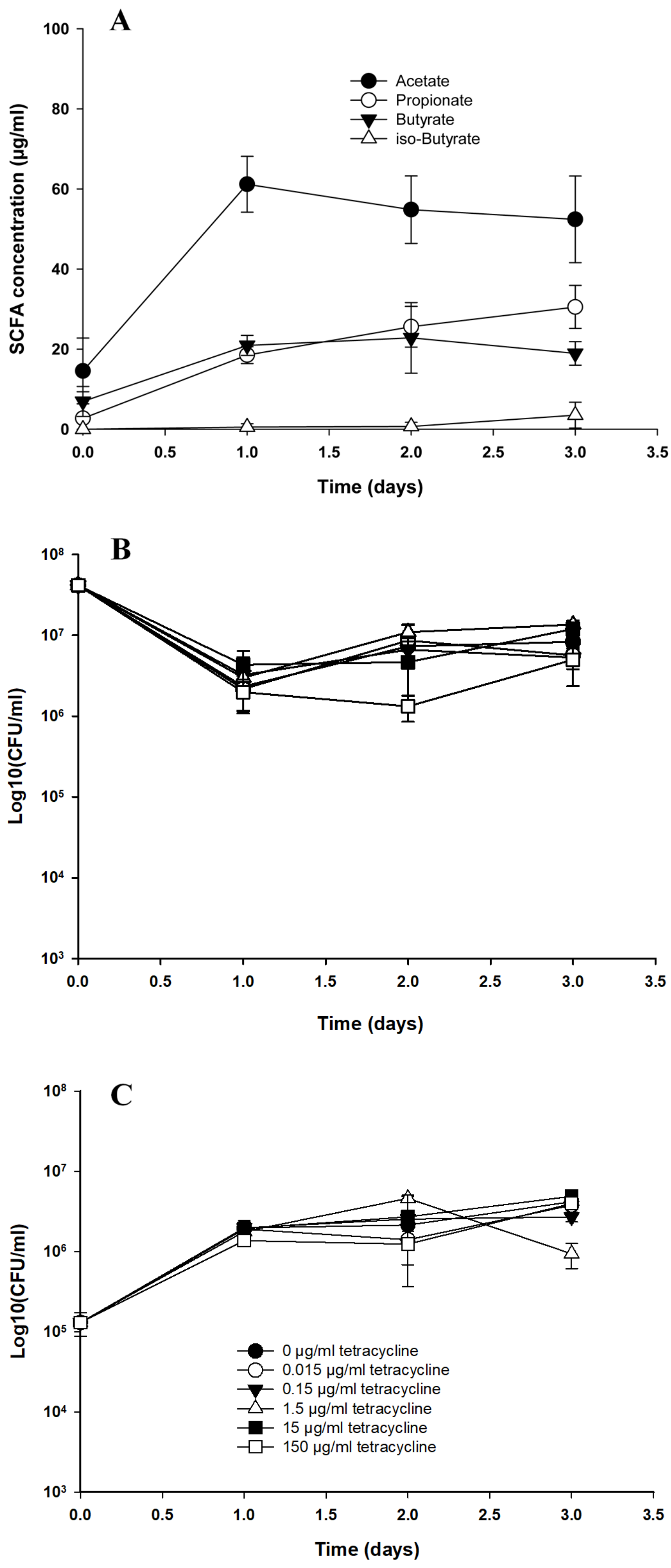

Figure S1. Plot of short chain fatty acid (SCFA) concentrations (A) and cell number on BHI (B) and CDC (C) for the six bioreactors over the course of the experiment.

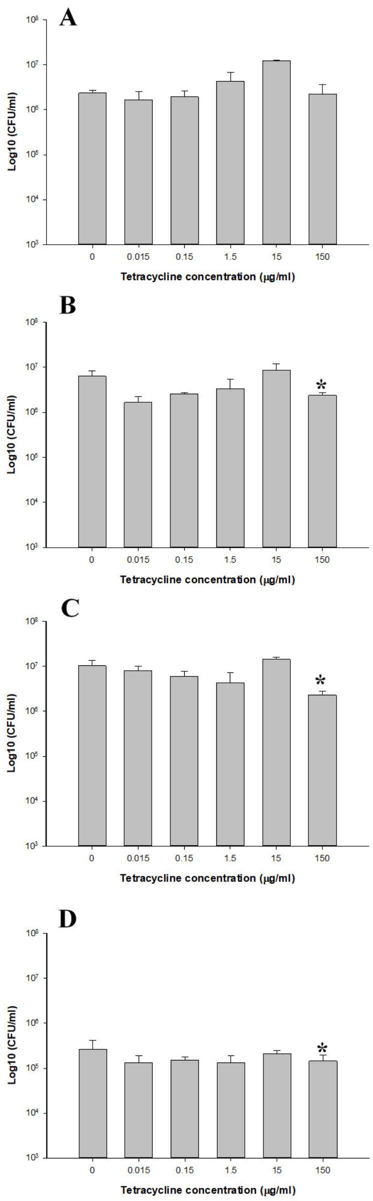

Figure S2. Comparison of *Fusobacterium*, *Lactobacillus*, *Eikenella corrodens* (betaproteobacteria), and *Bifidobacterium* viable counts on FSA (A), LMRS (B), CBA (C), and BIFIDO (D) in controls (no treatment) and tetracycline-treated samples after 7 days. *Indicates statistically significant differences from control ($p < 0.05$).
